# Continuous home cage monitoring of activity and sleep in mice during repeated paroxetine treatment and discontinuation

**DOI:** 10.1007/s00213-023-06442-3

**Published:** 2023-08-16

**Authors:** Helen M. Collins, Raquel Pinacho, S. K. Eric Tam, Trevor Sharp, David M. Bannerman, Stuart N. Peirson

**Affiliations:** 1 University Department of Pharmacology, Oxford, UK; 2 University Department of Experimental Psychology, Oxford, UK; 3 Sleep and Circadian Neuroscience Institute (SCNi), Nuffield Department of Clinical Neurosciences, Kavli Institute for Nanoscience Discovery, Dorothy Crowfoot Hodgkin Building, South Parks Road, Oxford, OX1 3QU UK

**Keywords:** SSRI, Paroxetine, Mice, Home cage monitoring, Non-invasive, Sleep, Circadian rhythms

## Abstract

**Rationale:**

Non-invasive home cage monitoring is emerging as a valuable tool to assess the effects of experimental interventions on mouse behaviour. A field in which these techniques may prove useful is the study of repeated selective serotonin reuptake inhibitor (SSRI) treatment and discontinuation. SSRI discontinuation syndrome is an under-researched condition that includes the emergence of sleep disturbances following treatment cessation.

**Objectives:**

We used passive infrared (PIR) monitoring to investigate changes in activity, sleep, and circadian rhythms during repeated treatment with the SSRI paroxetine and its discontinuation in mice.

**Methods:**

Male mice received paroxetine (10 mg/kg/day, *s.c.*) for 12 days, then were swapped to saline injections for a 13 day discontinuation period and compared to mice that received saline injections throughout. Mice were continuously tracked using the Continuous Open Mouse Phenotyping of Activity and Sleep Status (COMPASS) system.

**Results:**

Repeated paroxetine treatment reduced activity and increased behaviourally-defined sleep in the dark phase. These effects recovered to saline-control levels within 24 h of paroxetine cessation, yet there was also evidence of a lengthening of sleep bouts in the dark phase for up to a week following discontinuation.

**Conclusions:**

This study provides the first example of how continuous non-invasive home cage monitoring can be used to detect objective behavioural changes in activity and sleep during and after drug treatment in mice. These data suggest that effects of paroxetine administration reversed soon after its discontinuation but identified an emergent change in sleep bout duration, which could be used as a biomarker in future preclinical studies to prevent or minimise SSRI discontinuation symptoms.

**Supplementary Information:**

The online version contains supplementary material available at 10.1007/s00213-023-06442-3.

## Introduction

Sleep and circadian rhythm disruption (SCRD) is highly comorbid with depression and many other mental health disorders. Major depressive disorder (MDD), for example, produces circadian rhythm disturbances (reviewed in Walker et al. 2020), increases rapid eye movement (REM) sleep (Palagini et al. 2013) and causes insomnia in approximately 60% of patients (Geoffroy et al. 2018). Many psychoactive drugs also affect sleep, including antidepressants. Selective serotonin reuptake inhibitors (SSRIs) are currently the first-line pharmacological treatment of MDD and are known to promote wakefulness and suppress REM sleep by as much as 85% (Argyropoulos et al. 2009; McCarthy et al. 2016; Palagini et al. 2013; Saletu et al. 1991; Staner et al. 1995; Trivedi et al. 1999; Wichniak et al. 2017). Similar effects have been observed in preclinical studies of the SSRI paroxetine (Gervasoni et al. 2002; Kantor et al. 2017; Monaca et al. 2003; Neckelmann et al. 1996). In this way, SSRIs counteract increased sleep, one of the SCRD hallmarks of a depressive episode.

Nonetheless, SSRIs also have negative effects on sleep. Insomnia is a common side effect of SSRI therapy, especially at the beginning of treatment (Hickie et al. 2013; van Bemmel et al. 1993; Wilson and Argyropoulos 2005). SSRIs can also disrupt circadian rhythms, for example causing phase advancement in the activity of the suprachiasmatic nucleus (SCN), the mammalian “master clock” in the hypothalamus, as observed in rats in vivo and ex vivo (Nomura et al. 2008; Prosser et al. 1990; Sprouse et al. 2004, 2006).

Stopping treatment with an SSRI can also produce sleep disturbances in patients, including initial and middle insomnia, interrupted sleep, and vivid dreams (Barr et al. 1994; Black et al. 1993; Coupland et al. 1996; Davies and Read 2019; Dominguez and Goodnick 1995; Fava et al. 2015; Fava and Grandi 1995; Haddad 1997; Haddad et al. 1998; Jha et al. 2018; Louie et al. 1994; Mallya et al. 1993; Zajecka et al. 1998). Clinical trials have demonstrated that insomnia and sleeping difficulties occur in a significantly greater proportion of patients stopping SSRI medications than those maintained on-drug (Baldwin et al. 2006; Coupland et al. 1996; Fava et al. 2007; Hindmarch et al. 2000; Judge et al. 2002; van Geffen et al. 2005). Human electroencephalogram (EEG) studies have also shown that SSRI discontinuation can produce a rebound increase in REM sleep (Staner et al. 1995). Moreover, daytime somnolence, or the desire to go to sleep during the day, has also been reported in patients undergoing SSRI discontinuation (Black et al. 1993; Haddad et al. 1998; Zajecka et al. 1998).

Despite the well-documented existence of these symptoms, preclinical data on SSRI discontinuation are limited. For instance, three weeks of paroxetine treatment was shown to suppress REM sleep in rats, yet this had reversed within 24 h of discontinuation (Gervasoni et al. 2002). Similarly, the time that mice spent in both REM and non-REM (NREM) sleep did not differ from vehicle controls when EEG was performed two weeks after discontinuation from chronic paroxetine treatment (Kantor et al. 2017). SSRI-induced changes in sleep therefore appear to rapidly reverse following treatment cessation; yet these studies only measured sleep at two discrete time points, and how sleep changes over time during and after paroxetine administration has not been studied. SSRI treatment and discontinuation are therefore interesting examples of where continuous preclinical monitoring may be a useful way to assess changes in sleep/wake behaviour over time.

Home cage activity monitoring in mice provides a valuable tool to assess the effects of experimental interventions on physiology and behaviour. While cortical EEG provides the gold standard for determining sleep state in rodents, several approaches exist to continuously and non-invasively monitor mouse sleep. These include video recording (Fisher et al. 2012), piezoelectric sensors on the cage floor (Flores et al. 2007) and infrared beam break boxes (Pack et al. 2007). Recently, the Continuous Open Mouse Phenotyping of Activity and Sleep Status (COMPASS) system was developed as an alternative approach to simultaneously measure circadian rhythms in locomotor activity as well as sleep (Brown et al. 2016; Tam et al. 2021). COMPASS uses passive infrared (PIR) measurements to determine the amount and distribution of sleep as well as common measures of circadian stability (Brown et al. 2019). This approach is ideally suited for tracking changes in behaviour over extended periods of time, such as in disease models or during chronic drug administration.

Recently, we developed a model of paroxetine discontinuation in mice. We found that two days after discontinuation from repeated paroxetine administration, male mice showed evidence of increased anxiety-like behaviour compared to continued paroxetine and saline controls (Collins et al. 2022), mirroring another symptom of SSRI discontinuation in patients (Davies and Read 2019; Fava et al. 2015). This model therefore provides the opportunity to study sleep and circadian disturbances in mice known to exhibit other behavioural correlates of the human discontinuation syndrome. Here, we used PIR monitoring to simultaneously assess the effect of repeated paroxetine treatment and its discontinuation on home cage activity, sleep, and circadian rhythms in mice. A secondary aim of this study was to investigate how continuous home cage monitoring can be used to investigate the impact of long-term drug administration on mouse behaviour.

## Materials and methods

### Animals

Twenty four C57BL/6 J male mice (7 weeks old, Charles River) were single housed in large opaque open-top cages lined with sawdust. Female mice were not used as previous experiments suggested that behavioural effects of paroxetine discontinuation were evident in male but not female mice (Collins et al. 2022). Cages contained a small amount of sizzle nest bedding and mice had *ad* libitum access to food and water. Mice were housed at 21 °C on a 12:12 light–dark cycle (200 lx white LED light) in light tight chambers (LTC, 6 mice per LTC) for seven days before the start of the experiment. Experiments followed the principles of the Animal Research: Reporting of In Vivo Experiments (ARRIVE 2.0) guidelines and were conducted according to the United Kingdom Animals (Scientific Procedures) Act of 1986, under project license P6F11BC25 at the University of Oxford.

### Experimental design

**Fig. 1:**
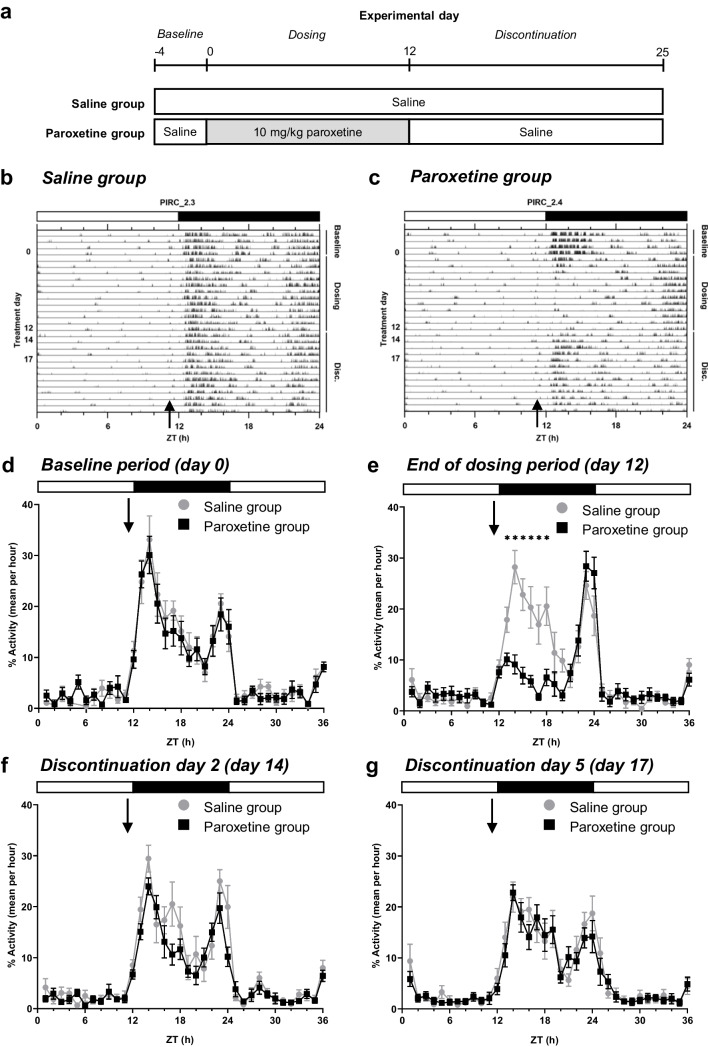
Effect of continued paroxetine and discontinuation on hourly activity. Experimental design (**a**). Representative actograms of a mouse in the Saline group (**b**) and the Paroxetine group (**c**). Each row of the actograms represents 24 h of activity (zeitgeist time, ZT), each spike represents activity (arbitrary units). Points represent mean ± SEM values for hourly activity comparing the in the Saline group and the Paroxetine group during the baseline period (treatment day 0) (**d**), at the end of paroxetine treatment (treatment day 12) (**e**), discontinuation day 2 (treatment day 17) (**f**) and discontinuation day 5 (treatment day 19) (**g**). Horizontal white bar represents light phase (05:00 to 17:00), black bar represents dark phase (17:00 to 05:00). Arrow represents time of daily injections. Saline group (n = 12), Paroxetine group (n = 12). Analysed with repeated measures ANOVA with post-hoc Bonferroni’s test (only the first 24 h included in analysis), * p < 0.05 between-subject comparisons (d-g)

Mice were allocated to one of two experimental groups by stratified randomisation: i) saline group: saline (0.9% sodium chloride) injections throughout; ii) paroxetine group: paroxetine injections (paroxetine hydrochloride, Abcam ab120069, 1 mg/ml in saline) during dosing period + saline during discontinuation period (Fig. 1a). Mice were handled for three days to habituate them to daily treatment. Mice were weighed daily throughout the experiment, with a humane endpoint set at 85% of baseline body weight. Mice were weighed and injections were given between 16:00 and 17:00 each day. The order in which mice were injected was varied each day to avoid confounds of treatment order.

All mice received once-daily 10 ml/kg *s.c.* saline injections for four days to establish a baseline weight and activity levels (baseline period, treatment day 0 represents the mean value for each mouse in the baseline period; Fig. 1a). Mice then received 12 days of either once-daily *s.c.* injections of saline or 10 mg/kg paroxetine. Paroxetine was chosen as it is the SSRI most likely to cause discontinuation symptoms in patients (Gastaldon et al. 2022; Price et al. 1996). The dose, frequency and duration of paroxetine administration were optimised in Collins et al. 2022, and reflect common doses and durations of SSRI treatment in the literature (Elizalde et al. 2008; Karlsson et al. 2011; Venzala et al. 2012). Doses of 5–10 mg/kg have also been shown to produce a serotonin reuptake transporter (SERT) occupancy of 80–92% in rodents (for example, Leiser et al. 2015; Severino et al. 2018), similar to the approximately 80% occupancy required for clinical therapeutic effects in patients (Meyer et al. 2004; Sanchez et al. 2014). *S.c.* injections were used rather than a more refined or continuous method of drug treatment (e.g., osmotic minipumps or drinking water administration) due to the limited solubility and bitter taste of paroxetine. This dosing period was then followed by a 13 day discontinuation period, where all mice received saline injections (Fig. 1a).

An a priori power calculation from data in a previous study suggested that n = 12/group was needed to detect significant effects of SSRI discontinuation on anxiety-like behaviour (power = 0.8, α = 0.05, expected effect size = 0.55, based on analysis with one-way ANOVA; Collins et al. 2022). In an initial cohort (n = 6/group), all mice received saline injections for seven days to monitor the effects of paroxetine discontinuation. Following preliminary evidence of persistent changes to sleep structure seven days after discontinuation, the discontinuation period was extended to 13 days in the second cohort to identify when such behavioural changes normalised to control levels (n = 12/group total) (Fig. 1a).

### Activity and sleep screening with PIR monitoring

Activity was automatically tracked with passive infrared sensors (Panasonic, AMN32111 NaPiOn) as in (Brown et al. 2016). Briefly, activity was detected every 100 ms (% time active reported in 10 s intervals) and plotted as actograms using a freely available function (https://github.com/bradmonk/actograph).

Data were binned into 1 h epochs using scripts written in MATLAB R2021b to calculate the mean hourly activity per day. Data were then averaged over each day, dark phase, and light phase. The interday stability (IS, the variability in activity of a 24 h period compared to the total variance of the whole experiment) and intraday variability (IV, a measure of the stability of a mouse’s sleep and wake periods) were calculated from activity data for each mouse as described in (Brown et al. 2019).

Behaviourally-defined sleep was defined as ≥ 40 s of immobility, which was previously validated using EEG and beam-break methods (Brown et al. 2016; Fisher et al. 2012; Pack et al. 2007), including recently in a mouse model of sleep disruption (Krone et al. 2021). Sleep data were binned into 1 h epochs to calculate the percentage of time asleep per hour, then averaged over each day, dark phase, and light phase per mouse. The latency to the first sleep bout after the lights turned on was calculated for each mouse (assigned a value of 0 s if the mouse was already asleep when the light was turned on). The number of sleep bouts in each dark and light phase were counted. The duration of each sleep bout was calculated and durations grouped into bins of ≤ 1 min, > 1 but ≤ 10 min (1–10 min), > 10 min but ≤ 60 min (10–60 min) or > 60 min (Ang et al. 2021), and expressed as a percentage of the total number of sleep bouts in each dark phase.

### Statistical analysis

Hourly activity on representative treatment days (baseline period, day 0; end of repeated paroxetine treatment, day 12; discontinuation day 2, day 14; discontinuation day 5, day 17) were analysed with repeated measures ANOVA with Bonferroni’s post-hoc tests. These treatment days were based on experiments in Collins et al. (2022): these experiments found that there were significant effects on anxiety-like behaviour on discontinuation day 2 but had dissipated by day 5. These days were therefore specifically analysed to draw comparisons to the existing data on anxiety-like behaviour.

Mean activity, percentage of time asleep, sleep latency, number of sleep bouts, sleep bout durations and body weight were analysed using mixed effects models with Geisser-Greenhouse corrections and Bonferroni’s post-hoc tests for between-subject comparisons. Data from day 25 were not included in analyses of the light phase as mice were culled before 17:00 that day. IS and IV data were analysed with repeated measures ANOVA with Bonferroni’s post-hoc tests. Weight gain data were determined to be parametrically distributed with a D’Agostino Pearson’s test and were analysed with Student’s t-tests. All analyses were conducted by an experimenter blind to treatment groups. P values of p < 0.05 were considered significant.

## Results

### Repeated paroxetine treatment reduced mean activity in the first half of the dark phase

To establish baseline activity measurements, all mice initially received four days of saline injections (baseline period; Fig. 1a). The paroxetine group then received 12 days of 10 mg/kg *s.c.* paroxetine injections (dosing period), before swapping back to saline injections for a further 13 days (discontinuation period) (Collins et al. 2022), whereas the saline control group received saline injections throughout. Representative actograms showed that activity was similar each day throughout the experiment in a mouse from the saline group (Fig. 1b). In the paroxetine group, however, activity was reduced in the first half of the dark phase during the dosing period. This suppression reversed to control levels within two days of paroxetine discontinuation (Fig. 1c). Across both treatment groups, activity was low in the light phase, except for a spike in activity at ZT 11.5, corresponding to the daily injections.

Analysis of activity per hour on representative treatment days confirmed these observations. On the final day of the baseline period (day 0), as expected there was no significant difference in activity between the saline and paroxetine groups (main effect of treatment: F_(1,22)_ = 0.5702, p = 0.4582; ZT*treatment interaction: (F_(23,506)_ = 0.4073, p = 0.9992; Fig. 1d). To assess the effects of repeated paroxetine dosing, data were compared on the final day of the dosing period (day 12). On this day, there was evidence of reduced activity in the first 6 h of the dark phase in paroxetine-treated mice compared to saline controls (main effect of treatment: F_(1,22)_ = 5.821, p = 0.0246; ZT*treatment interaction: F_(23,506)_ = 6.728, p < 0.0001; significant post-hoc between-subject comparisons shown in Fig. 1e). On discontinuation day 2 (day 14), there was no overall difference between the treatment groups. Although there was a significant ZT*treatment interaction, there were no significant post-hoc differences at any individual time point, suggesting that the effect of paroxetine administration had already reversed to saline-control levels (main effect of treatment: F_(1,22)_ = 3.455, p = 0.0765; ZT*treatment interaction: F_(23,506)_ = 1.720, p = 0.0065; Fig. 1f). There was also no difference between the groups on discontinuation day 5 (day 17; main effect of treatment: F_(1,22)_ = 0.7610, p = 0.3925; ZT*interaction: F_(23,506)_ = 0.6333, p = 0.9523; Fig. 1g). Thus, repeated paroxetine treatment reduced home cage activity levels in the first half of the dark phase, but this rapidly normalised to saline-control levels following discontinuation.

### Repeated paroxetine reduced dark phase activity, which rapidly reversed following discontinuation

To understand how home cage activity changed day-to-day during the dosing and discontinuation periods, mean daily activity, and activity in both the light and dark phases, were then analysed. A mixed-effects model was used to compare the saline and paroxetine groups (between-subject comparisons), and to compare daily activity to the baseline period (day 0) and the end of paroxetine treatment (day 12) (within-subject comparisons).

**Fig. 2:**
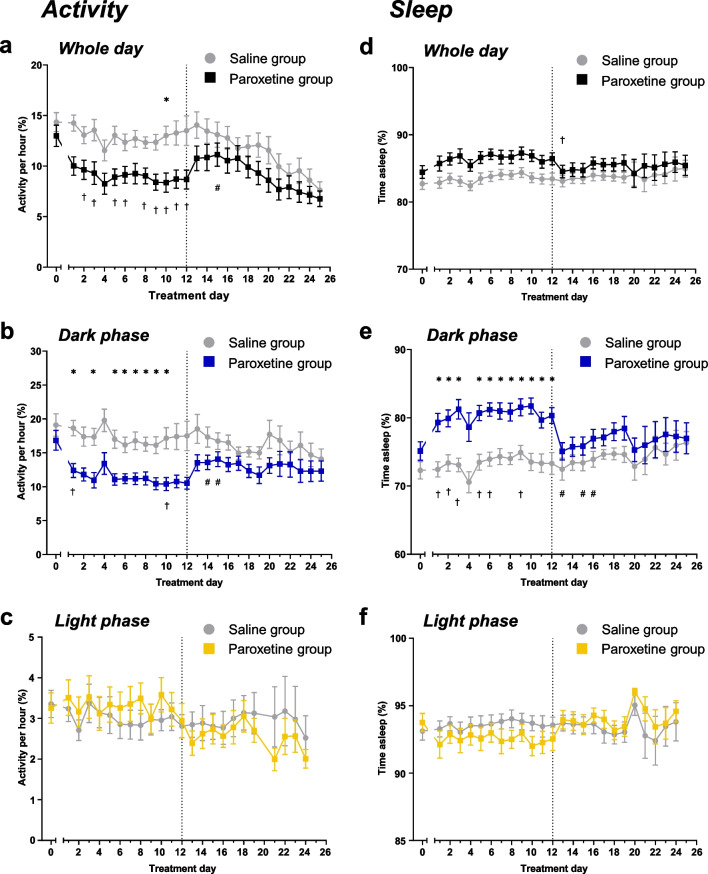
Effect of continued paroxetine and discontinuation on daily activity and sleep. Points represent mean ± SEM values of the percentage of time spent active across the whole day (**a**), dark phase (**b**) and light phase (**c**), and the percentage of time spent asleep across the whole day (**d**) dark phase (**e**) and light phase (**f**). Dotted vertical line represents discontinuation. Saline group (n = 12), Paroxetine group (n = 12), data from day 20 removed from light phase analysis due to outliers. Analysed with a mixed-effects model with Geisser-Greenhouse correction with post-hoc Bonferroni’s test, * p < 0.05 between-subject comparisons, † p < 0.05 day 0 vs 1–12, # p < 0.05 day 12 vs days 13–25 within-subject comparisons

Overall, continued paroxetine treatment reduced activity, but this effect reversed to control levels following discontinuation. There was a main effect of treatment group, as well as an effect of day and a treatment*day interaction (main effect of treatment: F_(1,22)_ = 4.993, p = 0.0359; effect of day: F_(25,550)_ = 6.242, p = 0.0013; interaction: F_(25,550)_ = 2.782, p < 0.0001; Fig. 2a). Post-hoc analyses showed that paroxetine administration significantly reduced activity compared to saline controls and the baseline period. Following paroxetine discontinuation, activity increased compared to the final day of paroxetine administration, but it was not significantly different to the baseline period or that of saline controls (Fig. 2a).

This reduction in activity was particularly apparent during the dark phase. There was an overall effect of treatment, as well as an effect of day and a significant treatment*day interaction (main effect of treatment: F_(1,22)_ = 8.144, p = 0.0092; effect of day: F_(25,550)_ = 5.012, p = 0.0031; interaction: F_(25,550)_ = 3.240, p < 0.0001; Fig. 2b). Post-hoc tests again showed that paroxetine administration reduced activity compared to saline controls and the baseline period. Activity increased following paroxetine discontinuation but did not differ from saline controls or the baseline period at any time following discontinuation (Fig. 2b). In contrast to the dark phase, neither continued paroxetine treatment nor discontinuation altered activity in the light phase (effect of treatment: F_(1,22)_ < 0.0001, p = 0.9886; effect of day: F_(23,506)_ = 3.155, p = 0.0179; interaction F_(23,506)_ = 1.524, p = 0.5770; Fig. 2c).

In summary, repeated SSRI treatment reduced mean daily activity, specifically during the dark phase. This effect was evident from the first day of paroxetine administration but returned to control levels within one day of discontinuation. There were no effects of paroxetine administration or cessation on activity during the light phase.

### Repeated paroxetine increased sleep, which also normalised following discontinuation

Behaviourally-defined sleep, defined as at least 40 s of immobility (Fisher et al. 2012; Pack et al. 2007) was then calculated from the activity data (Brown et al. 2016). There was no significant effect of treatment on the percentage of time mice spent asleep across the whole day, although there was a significant effect of day and a treatment*day interaction (main effect of treatment: F_(1,22)_ = 3.343, p = 0.0811; effect of day: F_(25,550)_ = 4.229, p = 0.0090; interaction: F_(25,550)_ = 2.159, p = 0.0011; Fig. 2d). Post-hoc tests did not find any between-subject differences, but there was a reduction in sleep from the end of repeated paroxetine treatment compared to the first day of discontinuation in the paroxetine group (Fig. 2d).

Paroxetine treatment increased the percentage of time mice spent asleep during the dark phase, which then normalised following discontinuation. There was a significant main effect of treatment, an effect of day and a treatment*day interaction (main effect of treatment: F_(1,22)_ = 5.373, p = 0.0301; effect of day: F_(25,550)_ = 5.887, p < 0.0001; interaction: F_(25,550)_ = 4.084, p < 0.0001; Fig. 2e). Post-hoc analyses showed that paroxetine administration significantly increased the percentage of time mice spent asleep compared to both saline controls and the baseline period (Fig. 2e). The percentage of time asleep fell following paroxetine cessation, and sleep did not differ from saline-treated controls or the baseline period on any day during the discontinuation period (Fig. 2e). In contrast, neither continued paroxetine treatment nor discontinuation altered the time spent asleep in the light phase (treatment: F_(1,22)_ = 0.0428, p = 0.8379; day: F_(24,528)_ = 3.496, p = 0.0115; interaction F_(24,528)_ = 2.518, p = 0.0001; Fig. 2f). Although there was a significant effect of day and a significant treatment*day interaction, there were no significant post-hoc differences between paroxetine-treated and saline-treated mice. There were also no differences in sleep latency (the time between the start of the light phase and sleep onset) between the groups (Suppl. Fig. 1).

In summary, paroxetine treatment increased the duration of time mice spent asleep in the dark phase. This effect reversed following paroxetine discontinuation, and there were no effects of paroxetine administration or its subsequent cessation on sleep during the light phase.

### Paroxetine treatment altered circadian rhythms

**Fig. 3:**
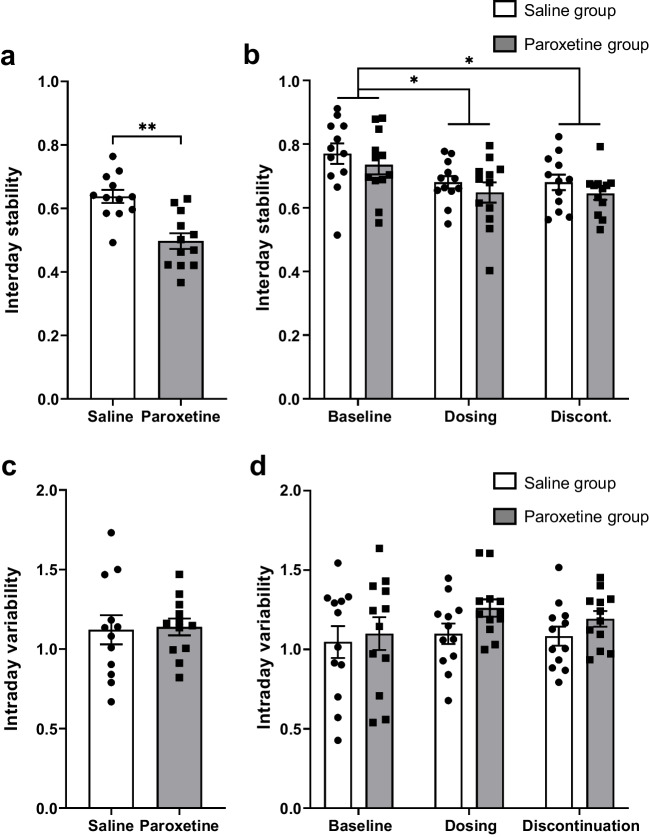
Effects of continued paroxetine and discontinuation on the stability of circadian rhythms. Bars represent mean ± SEM values of the interday stability (IS) (**a**) and IS by treatment period (**b**), and the intraday variability (IV) (**c**) and IV by treatment period (**d**). Saline group (n = 12), Paroxetine group (n = 12), dots represent individual mice. Analysed with Student’s t-tests (a, c) and repeated measures ANOVA with post-hoc Bonferroni’s test, ** p < 0.01

The amount and distribution of home cage activity can be used to measure circadian disruption using a range of different measures (Brown et al. 2019). To determine if paroxetine disrupted circadian rhythms, measures of the stability of circadian rhythms (interday stability, IS, and intraday variability, IV) were calculated from the daily activity data. Across the experiment (baseline, dosing, and discontinuation periods together), IS was significantly lower in the paroxetine group than in the saline group (t_(22)_ = 4.358, p = 0.0003; Fig. 3a), suggesting the activity patterns of the paroxetine-treated mice were less reproducible, and therefore less stable, than the saline-treated mice. When each treatment period was considered separately, IS was lower during the dosing and discontinuation periods compared to the baseline periods (effect of treatment period: F_(2,44)_ = 9.912, p = 0.0006; Fig. 3b), suggesting that activity became more variable as the experiment progressed. Nonetheless, there were no differences between paroxetine- and saline-treated mice in any individual phase (main effect of treatment: F_(1,22)_ = 1.587, p = 0.2209; treatment*period interaction: F_(2,44)_ = 0.0030, p = 0.9970; Fig. 3b), suggesting that activity patterns were similarly stable in both groups within each treatment period.

There was no difference in IV between the treatment groups across the whole experiment (t_(22)_ = 0.1730, p = 0.8640; Fig. 3c), or when the baseline, dosing and discontinuation periods were considered separately (main effect of treatment: F_(1,22)_ = 1.564, p = 0.2242; effect of treatment period: F_(2,44)_ = 1.983, p = 0.1582; treatment*period interaction: F_(2,44)_ = 0.5166, p = 0.6001; Fig. 3d). Thus, classical measures of circadian rhythms did not detect specific differences during repeated paroxetine treatment or its discontinuation.

### Paroxetine discontinuation lengthened sleep bouts in the dark phase

**Fig. 4:**
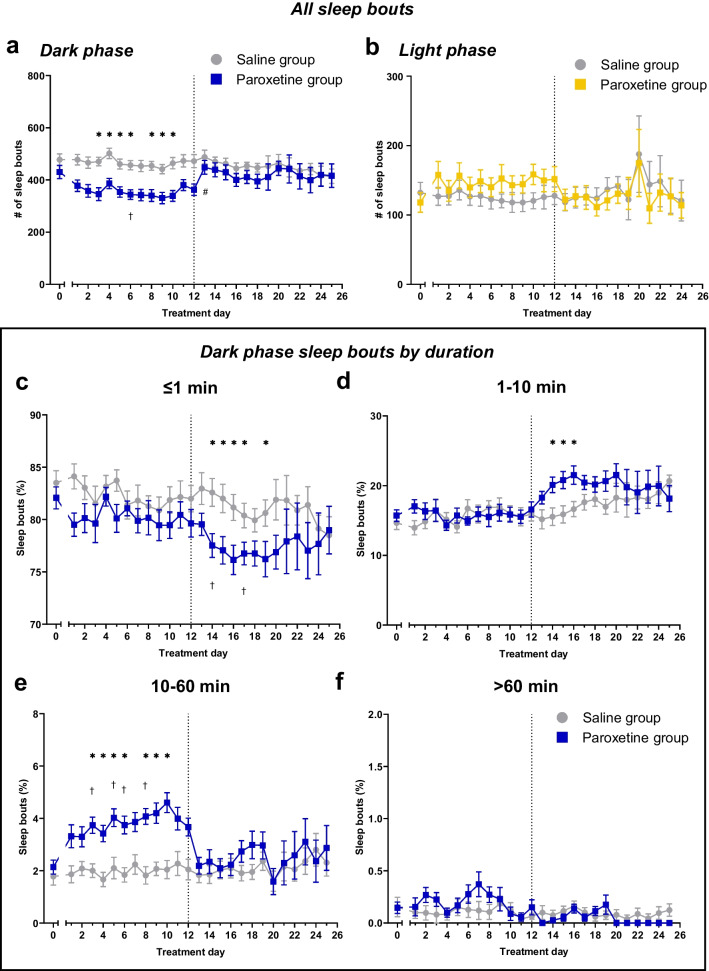
Effect of continued paroxetine and discontinuation on sleep bout distributions. Points represent mean ± SEM values of the total number of sleep bouts in the dark phase (**a**) and the light phase (**b**), and the percentage of sleep bouts of ≤ 1 min (**c**), 1–10 min (**d**), 10–60 min (**e**) and > 60 min duration (**f**) in the dark phase. Dotted vertical line represents discontinuation. Saline group (n = 12), Paroxetine group (n = 12). Analysed with a mixed-effects model with Geisser-Greenhouse correction with post-hoc Bonferroni’s test, * p < 0.05 between-subject comparisons, † p < 0.05 day 0 vs 1–12 within-subject comparisons

To investigate sleep fragmentation, the number of sleep bouts per dark and light phase were then calculated. In the dark phase, continued paroxetine treatment reduced the number of sleep bouts compared to saline controls. There was a significant main effect of treatment and a treatment*day interaction (main effect of treatment: F_(1,22)_ = 5.373, p = 0.0301; effect of day: F_(25,550)_ = 5.887, p < 0.0001; interaction: F_(25,550)_ = 4.084, p < 0.0001; Fig. 4a). Post-hoc tests showed that paroxetine administration reduced the number of sleep bouts compared to saline controls and the baseline period. The number of sleep bouts increased following paroxetine cessation but did not differ from saline controls or the baseline period on any day during the discontinuation period (Fig. 4a). By comparison, neither continued paroxetine nor discontinuation altered the number of sleep bouts in the light phase (F_(1,22)_ = 0.1342, p = 0.7176; day: F_(24,528)_ = 3.031, p = 0.0254; interaction: F_(24,528)_ = 1.750, p = 0.0145; Fig. 4b). Although there was a significant effect of day and a treatment*day interaction, there were no significant post-hoc differences between paroxetine- and saline-treated mice.

The fragmentation of sleep was then probed further by analysing the distribution of sleep bout durations in the dark and light phases. Mixed-effect models found that repeated paroxetine treatment did not alter the percentage of ≤ 1 min (Fig. 4c) or 1–10 min (Fig. 4d) sleep bouts, but it increased the proportion of sleep bouts of 10–60 min duration compared to both saline-treated controls and the baseline period (main effect of treatment: F_(1,22)_ = 6.730, p = 0.0166; effect of day: F_(25,550)_ = 5.184, p = 0.0010; interaction: F_(25,550)_ = 4.563, p < 0.0001; Fig. 4e). These findings are consistent with the reduction in the number of sleep bouts in the dark phase during paroxetine administration – mice were sleeping for longer and hence the number of bouts was lower.

Following paroxetine discontinuation, however, the percentage of 10–60 min sleep bouts immediately reduced back to saline-control and baseline levels (Fig. 4e). Moreover, there was an emergent reduction in the number of ≤ 1 min sleep bouts between the second and seventh days after discontinuation compared to both saline controls and the baseline period (main effect of treatment: F_(1,22)_ = 3.484, p = 0.0754; effect of day: F_(25,550)_ = 4.731, p = 0.0001; interaction: F_(25,550)_ = 1.466, p = 0.0495; Fig. 4c). Moreover, there was an increase in the percentage of 1–10 min sleep bouts between the second and fourth days after discontinuation compared to saline controls (main effect of treatment: F_(1,22)_ = 1.727, p = 0.2024; effect of day: F_(25,550)_ = 5.833, p < 0.0001; interaction: F_(25,550)_ = 2.785, p < 0.0001; Fig. 4d). Together, these results suggest a distribution of sleep bouts with fewer short sleep bouts in the dark phase in the week following paroxetine discontinuation, implying that sleep was less fragmented.

There were no significant effects of treatment or time, or a treatment*time interaction, on the percentage of > 60 min duration sleep bouts (treatment: F_(1,22)_ = 0.1036, p = 0.7506; time: F_(25,550)_ = 2.430, p = 0.3090; interaction: F(_25,550)_ = 1.742, p = 0.1521; Fig. 4f). Similar analysis was also conducted on the duration of sleep bouts in the light phase, but there were no effects of repeated paroxetine treatment or discontinuation (Suppl. Fig. 2).

### Paroxetine increased body weight, while discontinuation reduced weight gain

**Fig. 5:**
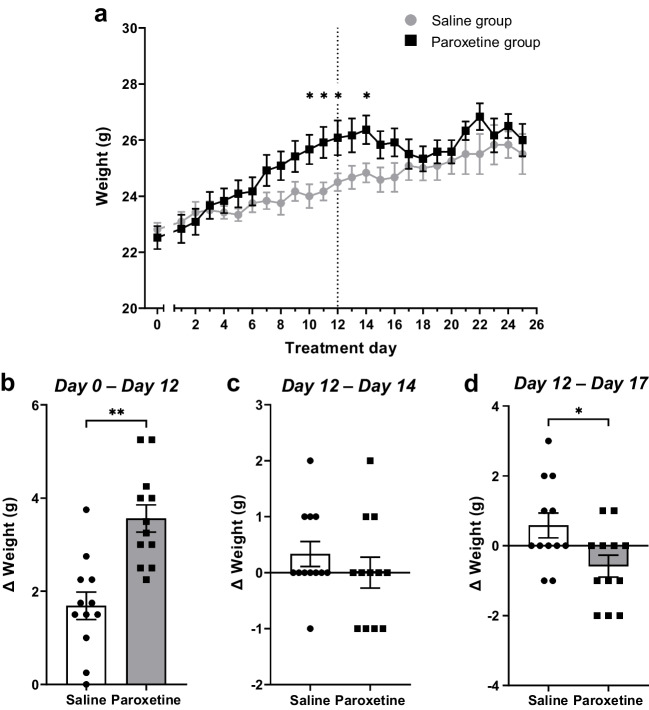
Effects of continued paroxetine and discontinuation on body weight. Points represent the mean ± SEM values for body weight each day (**a**). Dotted line represents paroxetine discontinuation. Bars represent the mean ± SEM values for weight change during paroxetine treatment (**b**), two days of discontinuation compared to the end of paroxetine treatment (**c**) and five days discontinuation compared to the end of paroxetine treatment (**d**). Saline group (n = 12), Paroxetine group (n = 12), dots represent individual mice. Mixed-effects model with Geisser-Greenhouse correction with post-hoc Bonferroni’s test (a) or Student’s t-test (b-d), * p < 0.05 ** p < 0.01 between-subject differences

The weight of the mice was also monitored throughout the experiment as an ongoing assessment of animal welfare. Overall, mice receiving paroxetine treatment gained more weight, whereas mice undergoing discontinuation gained less weight, than saline controls. All mice gained weight during the experiment (time: F_(25,550)_ = 32.05, p < 0.0001), in accordance with C57BL/6 mouse growth curves (Somerville et al. 2004). There was no effect of treatment but there was a significant treatment*day interaction (treatment: F_(1,22)_ = 1.110, p = 0.3036; interaction: F_(25,550)_ = 5.057, p < 0.0001; Fig. 5a). Between-subject comparisons found that mice receiving continued paroxetine weighed significantly more than saline-treated mice after repeated paroxetine administration (treatment days 10–14; Fig. 5a).

This increase in weight in paroxetine-treated mice was confirmed by comparing weight gain during the dosing period (t_(22)_ = 4.513, p = 0.0002 treatment day 0 vs day 12; Fig. 5b). By comparison, two days after paroxetine discontinuation, there was no difference in weight gain between the paroxetine and saline groups (day 12 vs 14: t_(22)_ = 0.9381, p = 0.3584; Fig. 5c). Five days after paroxetine discontinuation, mice had gained significantly less weight than saline controls (day 12 vs 17: t_(22)_ = 2.454, p = 0.0225; Fig. 5d). Thus, continued paroxetine increased weight gain, but mice gained less weight during discontinuation.

## Discussion

SSRI treatment and its subsequent discontinuation are known to affect sleep and circadian rhythms, and yet how these changes emerge and resolve during and after treatment have yet to be studied. Here, we investigated whether PIR monitoring could be used to detect changes in activity, sleep, and circadian rhythms in mice during repeated paroxetine treatment and its subsequent discontinuation. We found that paroxetine reduced activity in the dark phase compared to saline-treated controls, an effect that was particularly apparent in the first 6 h of the dark phase. Paroxetine administration was also associated with an increase in behaviourally-defined sleep during the dark phase compared to controls, and specifically an increase in the proportion of sleep bouts of 10–60 min duration. The effects of continuous paroxetine treatment reversed within 24 h of discontinuation, but there was also evidence of a reduction in the number of < 1 min sleep bouts and an increase in the number of 1–10 min sleep episodes, suggesting a lengthening of sleep bouts.

To our knowledge, non-invasive home cage monitoring has not previously been used to assess such sleep behaviour during chronic drug administration. Saline-treated mice in the current study provide a control for the effect of repeated systemic injections on spontaneous activity and sleep. Here, the daily activity patterns of the saline-treated mice were consistent throughout the experiment and were typical for wildtype C57BL/6 J mice (Pernold et al. 2021). For instance, the peak in activity early in the dark phase is a common feature of C57BL/6 behaviour and likely reflects initial dark phase activity and feeding following the inactive light phase (Hossain et al. 2004; Peirson et al. 2018). The second activity peak in the final few hours of the dark phase is also characteristic, and reflects that mice are typically most active around the light/dark transitions (Peirson et al. 2018). By comparison, activity was low and sleep was high in the light phase, as would be expected for a nocturnal species. These data confirm that the control mice did not have any underlying sleep disruption, which typically presents as increased activity in the light phase (Brown et al. 2019). Importantly, the interday stability and intraday variability values were similar to other reports in C57BL6 mice (Brown et al. 2019), suggesting that receiving daily saline injections did not disrupt the activity patterns of these mice. These results provide an important validation for the use of PIR monitoring for assessing circadian behaviours in chronic dosing studies of this kind.

In contrast to the saline control group, paroxetine administration had marked effects on activity and behaviourally-defined sleep in the dark phase. Paroxetine treatment reduced activity but increased sleep, with the number of sleep bouts decreasing but the proportion of longer sleep bouts increasing. These effects appeared after just one dose of paroxetine and were stable throughout the dosing period. Such activity-suppressant effects of paroxetine have also been reported in a mouse model of Huntington’s disease – repeated paroxetine treatment was associated with reduced activity in the dark phase compared to vehicle-treated controls, without changes to light phase behaviour (Kantor et al. 2017; Ouk et al. 2018). We also detected a reduction in spontaneous locomotor activity in mice treated for 28 days with paroxetine compared to saline controls (Collins et al. 2022).

Interestingly, the most robust reduction in activity by repeated paroxetine treatment was in the first 6 h after its administration. This finding mirrored the effect paroxetine produced in a previous study of circadian activity in rats (Gervasoni et al. 2002). The timing of this effect may reflect the short half-life of paroxetine (Kreilgaard et al. 2008), with the greatest effects occurring when its plasma, and ultimately brain, concentrations were at their highest. This altered activity pattern could be evidence of phase shifting and circadian disruption arising from elevated 5-HT in the SCN (Nomura et al. 2008; Sprouse et al. 2006, 2004). Alternatively, this reduction in activity could reflect the off-target blockade of noradrenaline reuptake transporters (NET) by paroxetine (Sanchez et al. 2014). Chronic administration of NET inhibitors has been shown to reduce spontaneous locomotor activity in mice (Mitchell et al. 2006). Hence, reduced locomotion could have arisen from the high plasma levels of paroxetine immediately following its injection, when its off-target effects will have been at their highest.

Paroxetine administration also increased sleep in the dark phase. This agrees with other studies suggesting that NREM increased in paroxetine-treated mice (Kantor et al. 2017) and rats (Leiser et al. 2015). On the other hand, this result was unexpected given that SSRIs often cause insomnia in patients (Thompson 2002; van Bemmel et al. 1993; Wilson and Argyropoulos 2005). Moreover, 5-HT transmission, which is elevated during SSRI treatment (Hajós-Korcsok et al. 2000; Malagié et al. 2000), is thought to promote wakefulness (McGinty and Harper 1976; Trulson and Jacobs 1979). For instance, electrophysiological recordings from juxtacellular-labelled 5-HT neurons in the rat DRN showed that firing progressively fell during the transition from wakefulness to NREM sleep (McGinty and Harper 1976). 5-HT is also thought to stimulate wakefulness by suppressing sleep-promoting nuclei (reviewed in Donlea et al. 2017). Moreover, genetic knockout studies have suggested that the 5-HT_1A_, 5-HT_2A_, 5-HT_2C_ and 5-HT_7_ receptors are critical regulators of sleep homeostasis (reviewed in Monti 2011). Increased extracellular 5-HT during paroxetine treatment would therefore be predicted to reduce sleep.

The present effect of repeated paroxetine therefore resembled the sedative effects of tricyclic antidepressants (Steriade 2004; Wichniak et al. 2017) and could have arisen from the off-target effects of paroxetine on cholinergic or noradrenergic transmission (Sanchez et al. 2014; Wilson and Argyropoulos 2005). While it is possible that reduced locomotor activity could have overestimated sleep, the sensitivity of the PIR system means that it can detect small movements associated with quiet wakefulness, such as grooming, even in the absence of locomotor activity. Thus, these data suggest that paroxetine both increased sleep and decreased locomotor activity when the animal was awake. Nonetheless, future EEG studies should be performed to confirm these findings.

Paroxetine discontinuation, on the other hand, was associated with a rapid reversal of the effects of continuous paroxetine treatment. Previous preclinical studies support these findings. For instance, the REM-suppressant effects of repeated paroxetine treatment reversed within 24 h of its washout in rats (Gervasoni et al. 2002), and NREM did not differ from vehicle controls two weeks after discontinuation of paroxetine in mice (Kantor et al. 2017). Thus, the current data suggest that the activity-suppressant and sleep-promoting effects of paroxetine reversed rapidly following treatment cessation.

In contrast, it was also predicted that there would be ongoing changes to sleep following discontinuation due to clinical observations of persistent insomnia and sleep disturbances. This discrepancy could reflect the difficulties comparing the effects of drugs that affect circadian rhythms in nocturnal rodents to diurnal humans. Alternatively, the short-lasting effects of paroxetine treatment may suggest that the dose, once-daily injections, or duration may not have been sufficient to produce discontinuation effects. Although the existing literature suggests that two weeks of SSRI administration produces steady state plasma levels within the clinical therapeutic range (Benmansour et al. 1999; Cremers et al. 2000), this treatment regime may not have led to the same neuroadaptive changes in the 5-HT system that occur clinically. Hence, the effects of paroxetine administration may in fact represent repeated, acute effects of paroxetine rather than adaptive changes, potentially explaining their rapid reversal following discontinuation. This may also have limited the extent of the subsequent discontinuation phenotype.

Despite the lack of clear parallels to the insomnia and sleep disturbances present in the clinical discontinuation syndrome, there was evidence that the structure of sleep bouts changed following paroxetine discontinuation in mice. In the dark phase, the duration of sleep bouts increased in length for seven days after the end of paroxetine treatment. This change in sleep bout duration could therefore act as a biomarker with which to further investigate SSRI discontinuation syndrome in rodents. These changes also occurred after paroxetine washout, and so unlike the potentially sedative effects of paroxetine itself, must have been distinct effects of discontinuation. Moreover, this duration of effect was substantially longer than the transient increase in anxiety-like behaviour previously seen only two days after paroxetine cessation (Collins et al. 2022), suggesting it may be a more translational marker of the syndrome.

The increase of dark phase sleep with longer sleep bouts could imply a weakened circadian drive for wakefulness, as observed in rats and squirrel monkeys with SCN lesions (Edgar et al. 1993; Moore 1983; Stephan and Zucker 1972), and in mice missing core clock genes (reviewed in Fisher et al. 2013). This change in sleep structure could also be likened to daytime somnolence, or patients feeling the desire to sleep more during the daytime (Black et al. 1993).

Alternatively, these data could be related to a rebound increase in REM sleep, which is reported to occur following SSRI cessation in patients. For example, one study found that paroxetine treatment suppressed REM sleep in humans compared to pre-treatment levels; within just two days of discontinuation, however, total REM sleep increased above pre-medication levels (Staner et al. 1995). REM rebound was also detected after fluoxetine and citalopram discontinuation (Feige et al. 2002; Trivedi et al. 1999; van Bemmel et al. 1993). For rodents, the probability of entering REM sleep grows as the length of the sleep bout increases (Kantor et al. 2017). Moreover, mice with REM deficits showed evidence of reduced sleep, suggesting changes in REM can alter overall sleep structure (Banks et al. 2020). By this logic, increased REM sleep could present as increased sleep bout durations, and therefore be an indication of discontinuation syndrome-like sleep disruption. Nonetheless, as the PIR system is not able to differentiate between sleep states, EEG studies would be needed to determine if REM rebound is detectable in this model of SSRI discontinuation.

Interestingly, there were no changes to light phase sleep or activity during paroxetine treatment or its discontinuation. Increased light phase activity is generally a hallmark of circadian disruption (Brown et al. 2019), so the lack of effect could suggest that neither paroxetine nor its discontinuation were associated with broad circadian disruption. On the other hand, the light/dark cycle that mice were housed under may have limited our ability to detect disruption. Light is the main cue to entrain sleep to the environment in rodents (Peirson et al. 2018), and can force sleep regardless of sleep pressure (Mrosovsky and Hattar 2003). Thus, evidence of sleep dysregulation may have been masked by the overwhelming drive to sleep during the light phase. Future experiments would therefore benefit from monitoring sleep and activity in constant free running conditions (continuous darkness), which may expose additional markers of sleep disturbances. The lack of effect of paroxetine on light phase activity and sleep may also relate to its half-life, meaning its greatest effects occurred during the dark phase immediately following administration. Continuous dosing methods such as osmotic minipumps could also be used to produce more consistent changes in behaviour across both the light and dark phase.

Continuously monitoring mice throughout paroxetine administration and discontinuation also made it possible to assess the effect of treatment on body weight. Increased weight gain has previously been reported in mice repeatedly treated with paroxetine (Zha et al. 2017, 2019). Moreover, reduced weight gain during paroxetine discontinuation could indicate increased stress, as weight loss and low appetite can be indicative of poor wellbeing in mice (Talbot et al. 2019). Alternatively, a lack of weight gain could indicate GI disruption, another common symptom of SSRI discontinuation syndrome (Fava et al. 2015). Thus, there may be ongoing changes to appetite, metabolism, or general wellbeing in the week following paroxetine discontinuation, potentially resembling to somatic symptoms of the discontinuation syndrome (Fava et al. 2015).

Our findings illustrate that PIR monitoring provides a valuable high-throughput screen of mouse activity and sleep in the weeks following paroxetine administration and discontinuation. The non-invasive nature of this approach means there is no need for surgical EEG electrode placement. This eliminates any potential inflammation resulting from neurosurgery and device implantation, which can produce changes in local neurotransmitter concentrations and alter behaviour (Albrecht et al. 2018; Balzekas et al. 2016). PIR monitoring can also be used over extended time periods, providing activity and sleep data in real time, rather than requiring extensive post hoc data analysis. Finally, this approach is advantageous over the behavioural tests previously used to assess changes in anxiety-like behaviour during SSRI discontinuation (Collins et al. 2022), in that more data can be obtained from fewer animals. Within-subject comparisons also increase the power of the study by reducing between-animal variability.

Nonetheless, this study did not include female mice. Previously, we failed to detect changes in anxiety-like behaviour in female mice undergoing paroxetine discontinuation (Collins et al. 2022); hence, this study exclusively used male mice. Although the explanation for this sex difference is not known, variation in the 5-HT system, for example differential SERT expression between male and female mice (Hodes et al. 2010), may contribute. Moreover, the faster metabolism of SSRIs in female compared to male rodents (Renoir et al. 2011) may limit their subsequent discontinuation effects. Given that women are more than twice as likely to take an SSRI than men (Wise 2014), future studies should optimise the administration of paroxetine to female mice to investigate the generalisability of the present findings to both sexes.

In conclusion, we found that repeated paroxetine treatment reduced activity but increased behaviourally-defined sleep in the dark phase, which reversed within 24 h of paroxetine cessation. While this dosing regimen did not recapitulate the common clinical side effects of SSRI treatment on sleep, there was evidence of an emergent lengthening of sleep bouts in the dark phase for up to a week following discontinuation that may relate to the symptom of daytime somnolence. There was also evidence of changes in weight gain at least five days after paroxetine cessation, again suggesting distinct effects of discontinuation. Changes in sleep structure could be used as objective biomarkers of the effects of SSRI discontinuation in mice. Thus, this paradigm provides the opportunity to investigate novel approaches to prevent the emergence of discontinuation symptoms, or to identify therapies that could minimise their severity, which could be translated to clinical studies. Our study also provides the first example of how non-invasive continuous home cage monitoring can detect changes in activity and sleep during repeated drug treatment in mice.

### Supplementary Information

Below is the link to the electronic supplementary material.

Supplementary file1 (DOCX 267 KB)

## Data Availability

All data produced in the manuscript can be made available upon reasonable request to the corresponding author.
