# Supplementary material for: Continuous home cage monitoring of activity and sleep in mice during repeated paroxetine treatment and discontinuation
Source: Psychopharmacology (Berl). 2023 Aug 16;240(11):2403–18. doi: 10.1007/s00213-023-06442-3 (PMC10593620; doi:10.1007/s00213-023-06442-3)
Supplement: Supplementary file 1 — Supplementary file1 (DOCX 267 KB) [file 213_2023_6442_MOESM1_ESM.docx]

## Supplementary information


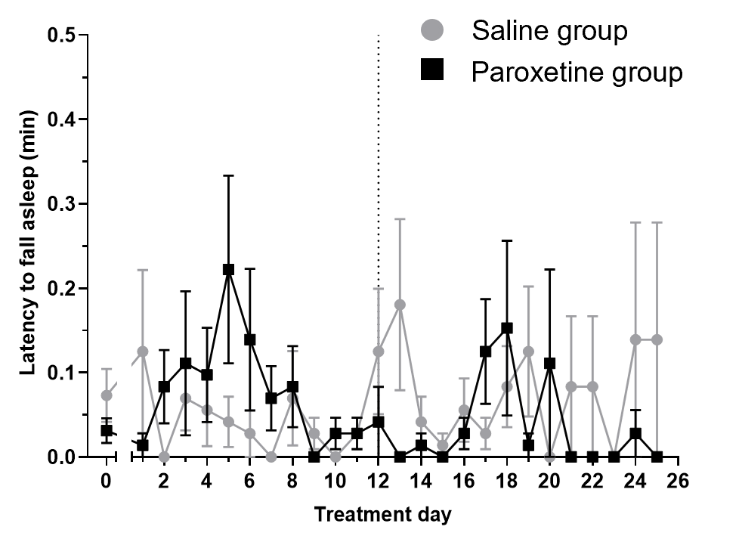


Suppl. Fig. 1 Effect of continued paroxetine and discontinuation on sleep latency. Points represent mean ± SEM values of the time between the start of the light phase and the first sleep bout for the saline and paroxetine groups. Dotted vertical line represents discontinuation. Saline group (n=12), Paroxetine group (n=12). Analysed with a mixed-effects model with Geisser-Greenhouse correction with post-hoc Bonferroni’s test (main effect of treatment: F_(1,22)_=0.0871, p=0.7706; effect of day: F_(25,550)_=0.8408, p=0.5643; interaction: F_(25,550)_=1.429, p=0.0835), as well as Mann Whitney U tests on representative treatment days (day 0 U=54.50, p=0.2945; day 12 U=59.50, p=0.3416; day 14 U=65.50, p=0.7391; day 17: U=57.00, p=0.2520).

**
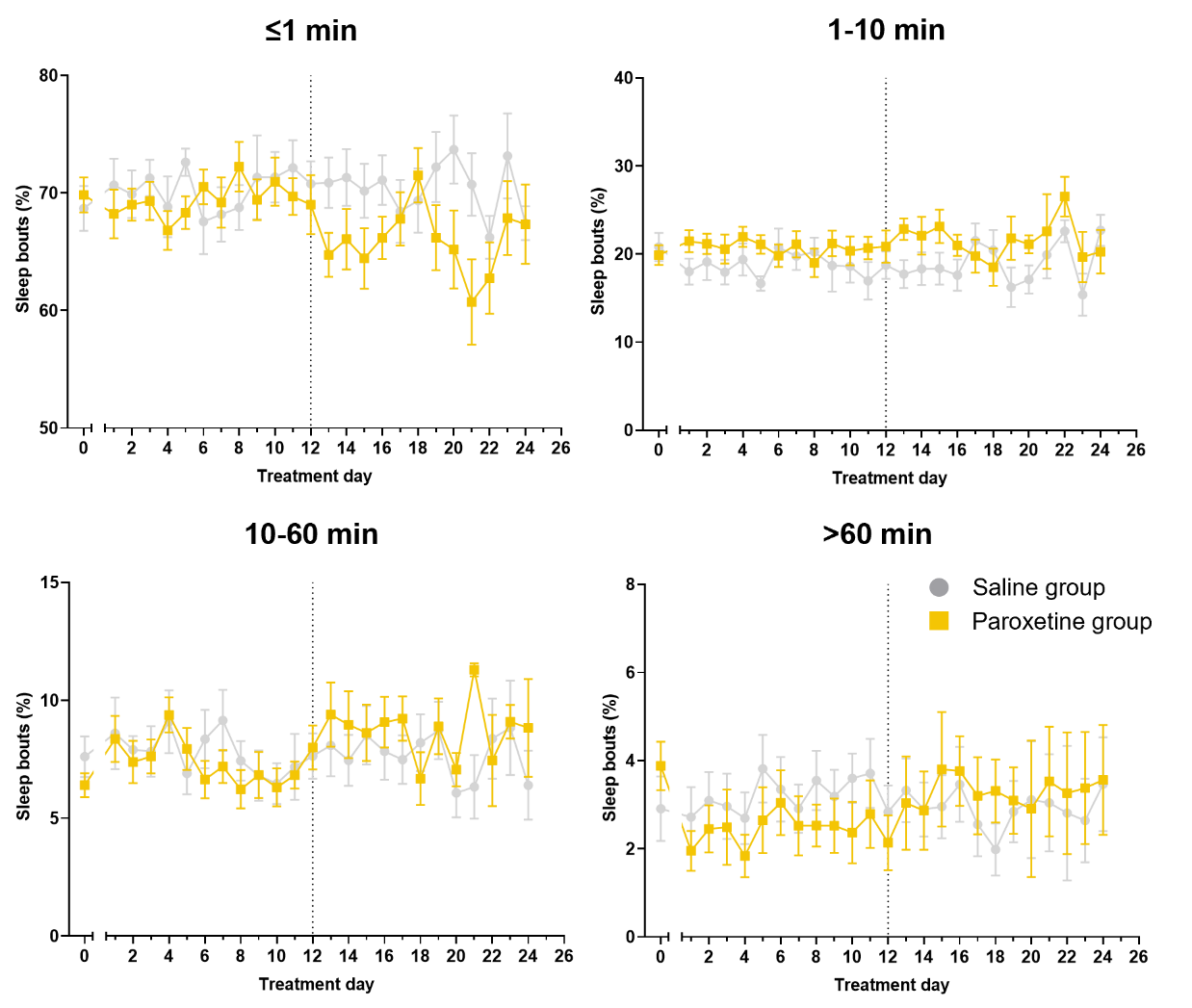
Suppl. Fig. 2 Effect of continued paroxetine and discontinuation on sleep bout duration distribution in the light phase.** Points represent mean ± SEM values of the percentage of sleep bouts of ≤1 min, 1-10 min, 10-60 min and >60 min duration in the light phase. Dotted vertical line represents discontinuation. Saline group (n=12), Paroxetine group (n=12). Analysed with a mixed-effects model with Geisser-Greenhouse correction with post-hoc Bonferroni’s test (<1 min, main effect of treatment: F_(1,22)_=1.843, p=0.1884; effect of day: F_(24,528)_=0.9318, p=0.5009; interaction: F_(24,528)_=1.242, p=0.1998; 1-10 min, main effect of treatment: F_(1,22)_=2.667, p=0.1167; effect of day: F_(24,528)_=0.7722, p=0.6392; interaction: F_(24,528)_=1.098, p=0.3416; 10‑60 min: main effect of treatment: F_(1,22)_=0.0014, p=0.9708; effect of day: F_(24,528)_=1.538, p=0.1341; interaction: F_(24,528)_=0.8730, p=0.6403; >60 min, main effect of treatment: F_(1,22)_=0.0066, p=0.9362; effect of day: F_(24,528)_=1.254, p=0.2923; interaction: F_(24,528)_=1.497, p=0.0624).
